# Estimating the short-time rate of change in the trend of the Keeling curve

**DOI:** 10.1038/s41598-020-77921-2

**Published:** 2020-12-04

**Authors:** Sven Nordebo, Muhammad Farhan Naeem, Pieter Tans

**Affiliations:** 1 Department of Physics and Electrical Engineering, Linnæus University, 351 95 Växjö, Sweden; 2 NOAA Global Monitoring Laboratory, Boulder, CO 80305 USA

**Keywords:** Climate sciences, Environmental sciences

## Abstract

What exactly is the short-time rate of change (growth rate) in the trend of $\text {CO}_2$ data such as the Keeling curve? The answer to this question will obviously depend very much on the duration in time over which the trend has been defined, as well as the smoothing technique that has been used. As an estimate of the short-time rate of change we propose to employ a very simple and robust definition of the trend based on a centered 1-year sliding data window for averaging and a corresponding centered 1-year difference (2-year data window) to estimate its rate of change. In this paper, we show that this simple strategy applied to weekly data of the Keeling curve (1974–2020) gives an estimated rate of change which is perfectly consistent with a more sophisticated regression analysis technique based on Taylor and Fourier series expansions. From a statistical analysis of the regression model and by using the Cramér–Rao lower bound, it is demonstrated that the relative error in the estimated rate of change is less than 5 $\%$. As an illustration, the estimates are finally compared to some other publicly available data regarding anthropogenic $\text {CO}_2$ emissions and natural phenomena such as the El Niño.

## Introduction

Are global events such as the natural climate phenomena, the major economic recessions or the COVID-19 global crisis and economic shutdown visible in the continuous monitoring of carbon dioxide in the atmosphere? This is a timely question that probably can not be given a definite answer, but which nevertheless is worthwhile to study and to discuss. This query also leads directly to the follow up question regarding how the short-time rate of change in the trend of ${\text {CO}}_2$ data can be defined and analysed.

The continuous monitoring of the atmospheric greenhouse gases as well as their historical, pre-industrial levels continues to provide an irreplaceable source of data, information and analyses that are becoming increasingly important for policymakers in their quest to mitigate the worst scenarios concerning the global warming, see e.g.^1–5^. To this end, continuous monitoring was started by David Keeling at NOAA’s Mauna Loa Observatory (MLO) in Hawaii, which gives access to well-mixed clean air at 3.5–4 km altitude. These measurements later became known as the “Keeling curve”, see e.g.^6^.

Trends and analyses of local as well as of global carbon dioxide cycles at various time-scales are continuously monitored and analysed by the National Oceanic and Atmospheric Administration (NOAA), see e.g. the curve fitting methods described in^1,7^. In this paper, we will revisit some of the basic regression analysis techniques and perform a statistical analysis based on weekly data from the Keeling curve covering the period from 1974-05-19 to 2020-03-29, cf.^8^ as well as the master’s thesis^9^. The purpose of this study is to validate a simple short-time estimate of the rate of change (or growth rate) in the trend of the ${\text {CO}}_2$ data. This simple, intuitive method is based on a centered 1-year data window to estimate the trend and a centered 1-year difference to estimate its rate of change. In fact, the latter can conveniently be interpreted as an estimation based on a differencing digital filter based on a 2-year data window. It is emphasized that the 1-year term is fundamental due to the annual periodicity of the natural global carbon dioxide cycle. It should be noted, however, that even though the simple digital filter approach takes full advantage of the 1-year term to create very effective spectral nulls, it also implies a limitation of the method in comparison to the more flexible regression techniques which can readily be adapted to any window length.

The simple digital filter approach turns out to be very stable and robust and could hence be used as a readily accessible standard definition of the short-time (1-year) trend and its rate of change. As a reference, the simple approach can always be validated by a comparison with any of the more sophisticated linear regression techniques that are available. In this study, we consider a well-established linear regression analysis based on 3 polynomial coefficients and 8 Fourier series coefficients (4 yearly harmonics) followed by smoothing (low-pass filtering) of the residuals, as is employed in^7^. The difference to the NOAA approach, however, is that we are using here a short-time data window of only 2 years and no postfiltering of the residuals. This enables us to perform a statistical analysis of the residual errors and to establish the corresponding estimation accuracy. We will demonstrate that the resulting residual errors based on a 2-year data window are in good agreement with the statistical assumption that the weekly data from 1974 to 2020 are corrupted by additive, uncorrelated Gaussian noise. Indeed, most of the daily variability in this data is caused by weather systems that bring different air masses to MLO so that there is memory from day to day, but not from week to week. There can also be variability on a seasonal scale, caused for example by droughts, or an early spring, etc., and which will then be seen as local changes in the short-time trend. We will show that the regression analysis that is considered here is perfectly consistent (within a standard deviation of $\pm \, 0.02$ ${\text{ppm/year}}$) with the simple estimation approach that has been described above. As a confirmation of our approach we have furthermore compared our results with the trend analyses performed by NOAA and found very good agreement provided that the smoothing of the residual data is adequately chosen.

The question about the smallest useful window length in the short-time estimate of the rate of change is an interesting, subtle issue. It is noted that the task to estimate the trend of the Keeling curve by averaging of continuous-time data is a well-posed problem. However, the task to differentiate the data is an ill-posed problem in the sense that the derivative does not depend continuously on the given data^10^. Hence, a regularization, or smoothing, is necessary. In a discrete-time linear regression analysis with a linear least squares (pseudo-inverse) solution, this ill-posedness will manifest itself as an increasingly ill-conditioned system matrix when the observation interval decreases. Obviously, the ill-conditioning also increases with an increasing number of regression parameters (such as a third derivative, etc). To obtain error estimates in such inverse problems, it is common to employ the Singular Value Decomposition (SVD) as well as very effective L-curve techniques where the residual error is plotted against some regularizing parameter^10–12^. Due to the Gaussian assumption referred to above, we can take a more direct approach here and consider the Maximum Likelihood (ML) estimation and the associated Cramér–Rao Lower Bound (CRLB)^13^ of the pertinent parameter as the objective for the L-curve analysis. The subsequent analysis will show that a stable estimate of the rate of change can be obtained with the 2-year window yielding a relative estimation error of less than 5 $\%$, see also^9^. Considering the long term data used in this study (1974–2020), it is finally worth noting that the error estimates that have been presented here are very much coherent with the $1.42\pm 0.02$ ${\text{ppm/year}}$ that was reported for the period 1974–1985, see^1^.

## A simple estimation strategy

Let $f_{{\text {CO}}_2}(j)$ denote the sequence of weekly data of the Keeling curve. We define the following estimate of the short-time trend

1 $$\begin{aligned} {\widetilde{f}}_{{\text {CO}}_2}(i)=\frac{1}{53}\sum _{j=i-26}^{i+26}f_{{\text {CO}}_2}(j), \end{aligned}$$

corresponding to a centered 1-year sliding window. It is noted that (1) can be interpreted as an approximation of the constant term in a Fourier series expansion of the 1-year data, or as the constant term in the corresponding 53-point Discrete Fourier Transform (DFT). Notice that the 53 week indices cover a time span of exactly 52 weeks (or 53 weeks in the sense of approximating an integral), which is a good approximation of 1 year. It is also desirable to employ an odd number of week indices to facilitate a symmetric window. The corresponding short-time rate of change can similarly be estimated from the following centered 1-year differences

2 $$\begin{aligned} \Delta _t {\widetilde{f}}_{{\text {CO}}_2}(i)={\widetilde{f}}_{{\text {CO}}_2}(i+26) -{\widetilde{f}}_{{\text {CO}}_2}(i-26) =\frac{1}{53}\left[ \sum _{j=i}^{i+52}f_{{\text {CO}}_2}(j) -\sum _{j=i-52}^{i}f_{{\text {CO}}_2}(j) \right] , \end{aligned}$$

which covers data over an interval of exactly 104 weeks (approximately 2 years).

From our empirical numerical experiments we have experienced that it is crucial to employ a symmetric data window centered in the middle of an observation interval in order to obtain reliable and consistent results^9^. Hence, the averaging (1) can be interpreted as a discrete-time (digital) filtering ${\widetilde{f}}_{{\text {CO}}_2}(i)=h(i)*f_{{\text {CO}}_2}(i)$ based on the symmetric (and non-causal) impulse response function

3 $$\begin{aligned} h(i)=\left\{ \begin{array}{ll} 1/53 &{} |i|\le 26, \\ 0 &{} {\text {otherwise}}, \end{array}\right. \end{aligned}$$

with the corresponding frequency response function

4 $$\begin{aligned} H(\nu )=\sum _{i}h(i){\text {e}}^{-{\text {j}}2\pi \nu i}=\frac{1}{53}\frac{\sin {\pi \nu 53}}{\sin (\pi \nu )}, \end{aligned}$$

and where $\nu$ is the frequency in ${{\text {weeks}}^{-1}}$. Note that (4) is the Discrete Time Fourier Transform (DTFT) defined for continuous frequencies^14^, p. 248, in contrast to the Discrete Fourier Transform (DFT) which is defined for uniformly sampled discrete frequencies^14^, p. 456. The DFT is usually computed by using an algorithm referred to as the Fast Fourier Transform (FFT). Similarly, the differencing (2) corresponds to a digital filtering $\Delta _t {\widetilde{f}}_{{\text {CO}}_2}(i)= g(i)*f_{{\text {CO}}_2}(i)$ with the anti-symmetric impulse response

5 $$\begin{aligned} g(i)=h(i+26)-h(i-26)=\left\{ \begin{array}{ll} 1/53 &{} 1\le i\le 52, \\ -1/53 &{} -52\le i\le -1, \\ 0 &{} {\text {otherwise}}, \end{array}\right. \end{aligned}$$

and frequency response function

6 $$\begin{aligned} G(\nu )=\sum _{i}g(i){\text {e}}^{-{\text {j}}2\pi \nu i}=H(\nu ){\text {e}}^{{\text {j}}2\pi \nu 26}-H(\nu ){\text {e}}^{-{\text {j}}2\pi \nu 26}={\text {j}}2H(\nu )\sin (\pi \nu 52). \end{aligned}$$

**Figure 1:**
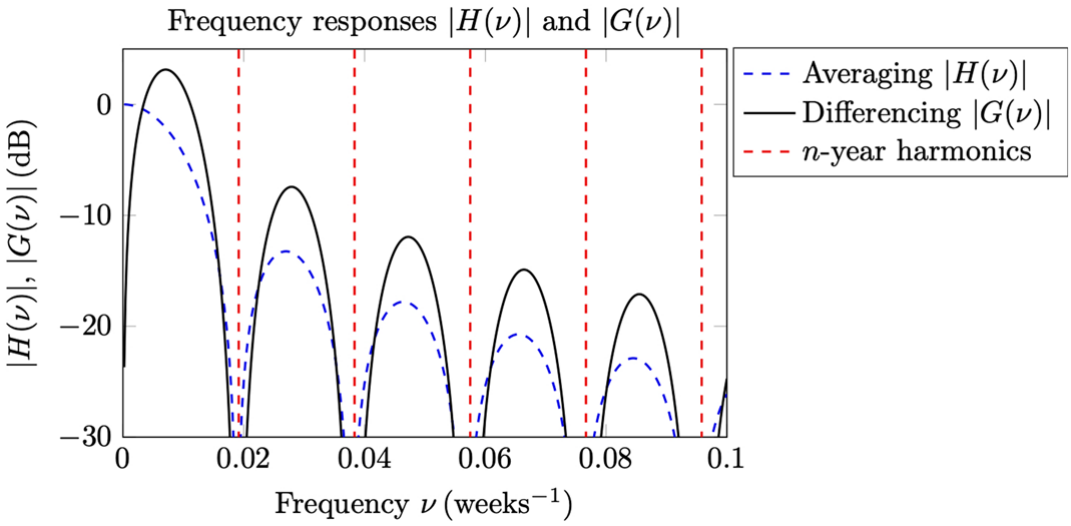
Magnitudes of the frequency responses of the digital averaging and differencing filters, $H(\nu )$ and $G(\nu )$, respectively. The frequency of the yearly harmonics for $n=1,\ldots ,5$ are indicated with the vertical dashed lines.

It is noted that $H(\nu )$ is a low-pass filter with spectral zeros at $\nu =m/53$ weeks^−1^ for integer $m\ne 0$. Similarly, $G(\nu )$ is a differentiating high-pass filter with additional spectral zeros at $\nu =m/52$ weeks^−1^ for all integers *m*. In Fig. 1 is plotted the magnitudes of the frequency responses $H(\nu )$ and $G(\nu )$. As can be seen in this figure, the ability of the filters to smooth and to remove the periodic annual variability in the data stems from the deep nulls that are created close to the yearly harmonics at *n*/52.1775 weeks^−1^, and where 1 year corresponds approximately to 52.1775 weeks. Note that the double zeros of $G(\nu )$ are at either side of these yearly harmonics. To validate the simple estimation strategy proposed in (1) and (2) above, a statistical regression model will be defined and analysed below, see also^1, 7, 9^.

## Statistical modeling

Define a time window of $\Delta T$ ${\text {years}}$ covering exactly *M* weeks ( $M+1$ week-points) and where *M* is an even integer. Let the time window be centered at time $t_i$ where *i* is a week-index and define the temporary time variable $\tau =t-t_i\in [-\Delta T/2,\Delta T/2]$ where *t* is the decimal time in years (e.g. starting at 0 some 2020 years ago). Let $f_{{\text {CO}}_2}(\tau _n)$ denote the correspondingly sampled weekly data of the Keeling curve at (translated) time $\tau _n\in [-\Delta T/2,\Delta T/2]$ for $n=1,\ldots ,M+1$.

Consider now the following statistical model

7 $$\begin{aligned} f_{{\text {CO}}_2}(\tau _n)=a_0+a_1\tau _n+\frac{a_2}{2}\tau _n^2 +\sum _{k=1}^{4}\left\{ b_k\cos (2\pi k\tau _n)+c_k\sin (2\pi k\tau _n)\right\} +w(\tau _n), \end{aligned}$$

where $n=1,\ldots ,M+1$ and where $a_j$, for $j=0,1,2$, are Taylor series coefficients defining the trend and its first and second derivative, and $b_k$ and $c_k$ the Fourier series coefficients modeling the annual changes. The remaining model error $w(\tau _n)$ is assumed to be zero mean uncorrelated Gaussian noise with variance $\sigma ^2$. The statistical model (7) is a linear regression model which is conveniently written in matrix form as

8 $$\begin{aligned} \mathbf{x}=\mathbf{A}{\varvec{\theta }}+\mathbf{w}, \end{aligned}$$

where $\mathbf{x}$ is an $(M+1)\times 1$ vector representing the Keeling curve ${\text {CO}}_2$ data, $\mathbf{A}$ is an $(M+1)\times P$ matrix representing the basis functions defined in (7) and ${\varvec{\theta }}$ is a $P\times 1$ vector representing the model parameters $a_0,a_1,a_2,b_1,\ldots ,b_4,c_1,\ldots ,c_4$. Here, we are using $P=11$ regression parameters as in^7^, but other model orders can easily be incorporated and investigated in a similar way. The covariance matrix of the noise is given by $\mathbf{C}=E\left\{ \mathbf{w} \mathbf{w}^{\text {T}}\right\} =\sigma ^2\mathbf{I}$ where $E\left\{ \cdot \right\}$ denotes the expectation operator, $(\cdot )^{\text {T}}$ the transpose and $\mathbf{I}$ the identity matrix.

The Fisher Information Matrix (FIM) and the Maximum-Likelikelihood (ML) estimate for this situation are given by

9 $$\begin{aligned} {{\mathscr {I}}}=\frac{1}{\sigma ^2}{} \mathbf{A}^{\text {T}}{} \mathbf{A}, \end{aligned}$$

and

10 $$\begin{aligned} \widehat{{\varvec{\theta }}}_{\text {ML}}(\mathbf{x})=\left( \mathbf{A}^{\text {T}}{} \mathbf{A} \right) ^{-1}{} \mathbf{A}^{\text {T}}{} \mathbf{x}, \end{aligned}$$

respectively, see e.g.^13^. The corresponding Cramér–Rao Lower Bound (CRLB) is given by

11 $$\begin{aligned} E\left\{ (\widehat{{\varvec{\theta }}}(\mathbf{x})-{\varvec{\theta }})(\widehat{{\varvec{\theta }}}(\mathbf{x})-{\varvec{\theta }})^{\text {T}} \right\} \ge {{\mathscr {I}}}^{-1} =\sigma ^2\left( \mathbf{A}^{\text {T}}\mathbf{A} \right) ^{-1}, \end{aligned}$$

which is valid for any unbiased estimator $\widehat{{\varvec{\theta }}}(\mathbf{x})$ of ${\varvec{\theta }}$. Since the statistical model (8) is linear and the noise is assumed to be Gaussian, the bound in (11) is in fact achieved and the ML-estimate (10) is said to be efficient, see^13^. Here, we are particularly interested in the Cramér–Rao lower bound of the two parameters $a_0$ (the trend of the Keeling curve) and its derivative $a_1$, given by

12 $$\begin{aligned} {\text {CRB}}\{a_0\}&= \left[ {{\mathscr {I}}}^{-1} \right] _{11}=\sigma ^2 \left[ \left( \mathbf{A}^{\text {T}}\mathbf{A}\right) ^{-1}\right] _{11}, \end{aligned}$$

13 $$\begin{aligned} {\text {CRB}}\{a_1\}&= \left[ {{\mathscr {I}}}^{-1} \right] _{22}=\sigma ^2 \left[ \left( \mathbf{A}^{\text {T}}\mathbf{A}\right) ^{-1}\right] _{22}, \end{aligned}$$

i.e. the first and second diagonal elements of ${{\mathscr {I}}}^{-1}$, respectively.

Define the residual error

14 $$\begin{aligned} {\varvec{\varepsilon }}=\mathbf{x}-\mathbf{A}\widehat{{\varvec{\theta }}}_{\text {ML}}(\mathbf{x})=\mathbf{P}{} \mathbf{x}, \end{aligned}$$

where $\mathbf{P}{} \mathbf{x}$ is the projection onto the nullspace of $\mathbf{A}^{\text {T}}$, and the projection matrix is $\mathbf{P}=\mathbf{I}-\mathbf{A}\left( \mathbf{A}^{\text {T}}{} \mathbf{A} \right) ^{-1}{} \mathbf{A}^{\text {T}}$. Based on (8), it is readily seen that $E\left\{ {\varvec{\varepsilon }} \right\} ={{\varvec{0}}}$, and

15 $$\begin{aligned} \mathbf{C}_\varepsilon =E\left\{ {\varvec{\varepsilon }}{\varvec{\varepsilon }}^{\text {T}} \right\} =\sigma ^2\mathbf{P}^2, \end{aligned}$$

where we have employed that $E\left\{ {\varvec{x}} \right\} =\mathbf{A}{\varvec{\theta }}$ and $\mathbf{P}{} \mathbf{A}=\mathbf{0}$. Based on (15), we see that the variance of the noise can be determined as

16 $$\begin{aligned} \sigma ^2=\frac{{\text {tr}}\left\{ \mathbf{C}_\varepsilon \right\} }{{\text {tr}}\left\{ \mathbf{P}^2 \right\} }, \end{aligned}$$

where ${\text {tr}}\left\{ \cdot \right\}$ denotes the trace of a matrix. It is finally emphasized that the results (9) through (13) depend on the Gaussian assumption, whereas the only requirement for (15) and (16) is that the least squares method (10) is employed and that the noise has zero mean.

## Evaluation of the statistical model

### Comparison of estimates

**Figure 2:**
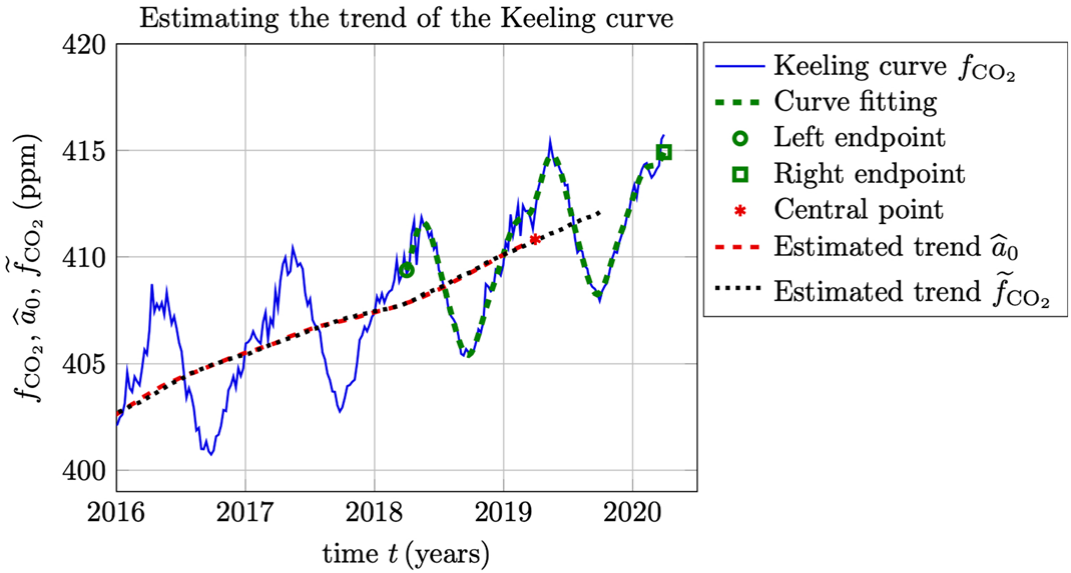
Estimating the trend of the Keeling curve. The estimate ${\widehat{a}}_0$ is based on (7) and (10) for a 2-year window and the estimate ${\widetilde{f}}_{{\text {CO}}_2}$ on a 1-year window (1). The left and right endpoints of the 2-year curve fitting are indicated with the circle and the square, respectively. The estimate ${\widehat{a}}_0$ at the central point of the window is indicated by the asterisk.

The estimates (10) can now be repeated based on an $M+1$ point sliding window defined by a sequence of weekly sampled central points $t_i$, as described above. In Fig. 2 is illustrated the Keeling curve based on noisy weekly data, together with the trend estimates ${\widehat{a}}_0$ using (10) for a $M=104$ week (2 year) window. The 1-year short-time trend ${\widetilde{f}}_{{\text {CO}}_2}$ defined in (1) is also included for comparison. The two estimates ${\widehat{a}}_0$ and ${\widetilde{f}}_{{\text {CO}}_2}$ are indistinguishable in this plot. It is noticed, however, that the estimate ${\widetilde{f}}_{{\text {CO}}_2}$ extends to half a year from the endpoint of the given data.

**Figure 3:**
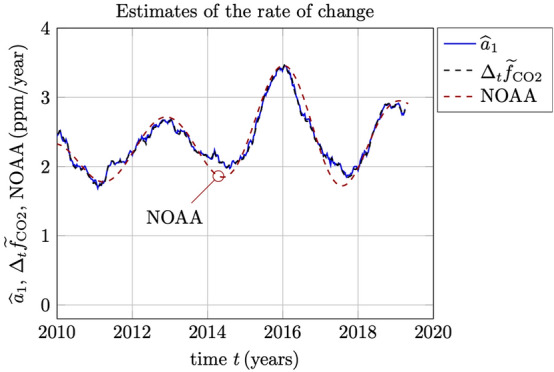
Estimates of the rate of change in the Keeling curve. Here, ${\widehat{a}}_1$ is based on the regression analysis (7) and (10), and $\Delta _t {\widetilde{f}}_{{\text {CO}}_2}$ is defined by the averaging (1) and differencing (2). These two estimates are indistinguishable in this plot. The red dashed plot (NOAA) is based on a long term trend and a 1.4 year smoothing chosen to fit our short-time estimates.

In Fig. 3 is plotted the two estimates of the rate of change ${\widehat{a}}_1$ using (10) for the $M=104$ week (2-year) window and $\Delta _t {\widetilde{f}}_{{\text {CO}}_2}$ defined in (2). As can be seen, the two estimates are indistinguishable in this plot. The estimated standard deviation in the error between the two estimates for the whole period 1974–2020, is 0.02 ${\text{ppm/year}}$. In Fig. 3 we have also included a comparison to the trend analysis performed by NOAA, and which is described in detail in^1,7^. The NOAA estimate shown here is based on a long-term trend and where a smoothing of the residual data has been employed with a Full Width at Half Maximum (FWHM) of 1.4 year to match the amplitude of the short-time estimates.

### Validation of the Gaussian assumption

**Figure 4:**
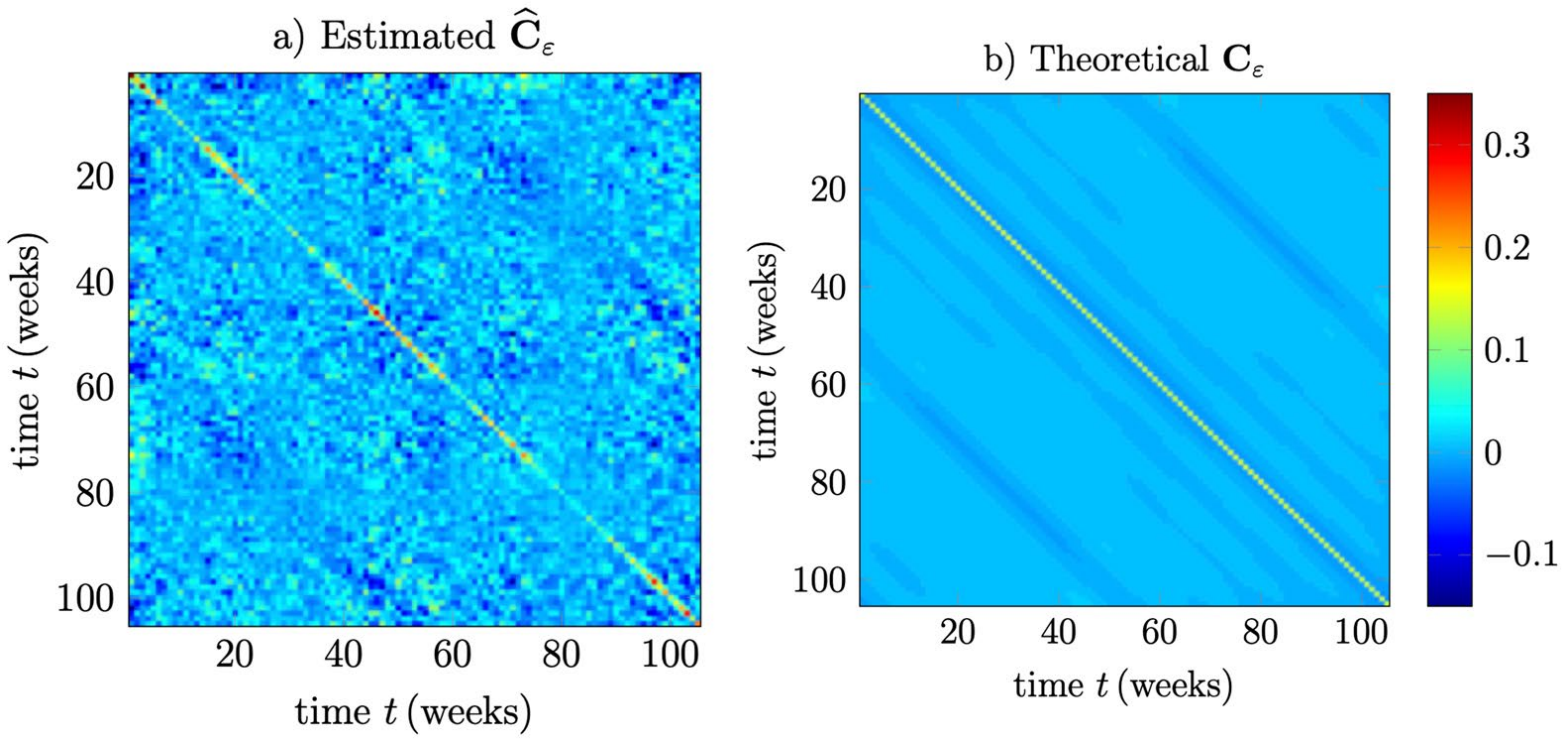
Estimated and theoretical values of the residual covariance matrix $\mathbf{C}_\varepsilon$. The theoretical values are calculated as $\widehat{\sigma ^2}{} \mathbf{P}^2$.

To validate the Gaussian assumption above, we perform a statistical analysis of the residual error ${\varvec{\varepsilon }}$ defined in (14). The 2-year (104 week) window is used once again and the covariance matrix $\mathbf{C}_\varepsilon$ defined in (15) is estimated by the sample covariance matrix

17 $$\begin{aligned} \widehat{\mathbf{C}}_\varepsilon =\frac{1}{N}\sum _{j=1}^{N} {\varvec{\varepsilon }}_j{\varvec{\varepsilon }}_j^{\text {T}}, \end{aligned}$$

where the sequence ${\varvec{\varepsilon }}_j$ consists of residual vectors of dimension $(M+1)\times 1$ sampled over $N=23$ non-overlapping 2-year intervals in the period 1974 to 2020 and where $M=104$. Based on (16), the variance of the noise can now be estimated as

18 $$\begin{aligned} \widehat{\sigma ^2}=\frac{{\text {tr}}\left\{ \widehat{\mathbf{C}}_\varepsilon \right\} }{{\text {tr}}\left\{ \mathbf{P}^2 \right\} }, \end{aligned}$$

giving the numerical value $\widehat{\sigma ^2}=0.145$ ${{\text {ppm}}{^2}}$. The resulting estimates are illustrated in Fig. 4a showing the values of $\widehat{\mathbf{C}}_\varepsilon$ in a comparison to the theoretical correlation values properly scaled as $\widehat{\sigma ^2}\mathbf{P}^2\sim \mathbf{C}_\varepsilon$ and which are plotted in Fig. 4b. The two plots in Fig. 4a,b are plotted in the same color range with max and min values according to $\widehat{\mathbf{C}}_\varepsilon$. As can be seen in these figures, the estimated as well as the theoretical covariance matrices have almost Toeplitz structures, indicating that the elements of ${\varvec{\varepsilon }}$ can be considered to be drawn from a stochastic process that is almost weekly stationary. The covariance matrices furthermore have a pronounced diagonal dominance indicating that the elements of ${\varvec{\varepsilon }}$ are almost (but not quite) uncorrelated, in accordance with (15).

**Figure 5:**
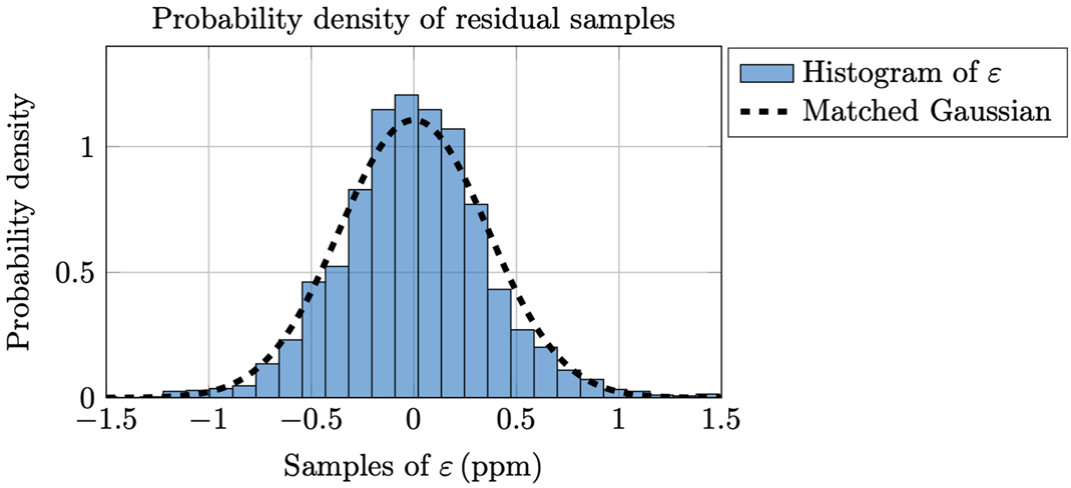
Probability density function for residual samples. The histogram is created by using 23 2-year (104 week) non-overlapping intervals from the period 1974–2020. The matching to the Gaussian density has been obtained with an estimated variance $\widehat{\sigma _\varepsilon ^2}=0.130$ ppm^2^.

Finally, we evaluate the Gaussian assumption by stacking all the $(M+1)\times 1$ non-overlapping residual vectors ${\varvec{\varepsilon }}_j$ together for $j=1,\ldots ,N$ where $N=23$ and $M=104$, and create the corresponding histogram. The corresponding samples are not completely uncorrelated, but they can be treated as being approximately uncorrelated due to the study illustrated in Fig. 4 above. We can furthermore assume that the residual samples are approximately identically distributed having the same variance, cf. the almost constant diagonal terms seen in Fig. 4. A normalized histogram over all of these residual samples are shown in Fig. 5 together with the theoretical Gaussian density corresponding to an estimated variance obtained here as $\widehat{\sigma _\varepsilon ^2}=0.130$ ppm^2^. For $M=104$ weeks we have ${\text {tr}}\left\{ \mathbf{P}^2 \right\} =94$, and following (18) we obtain here $\widehat{\sigma ^2}=\widehat{\sigma _\varepsilon ^2}(M+1)/{\text {tr}}\left\{ \mathbf{P}^2 \right\} =0.145$ ppm^2^, and which agrees perfectly with the previously obtained value.

### Sensitivity analysis

**Figure 6:**
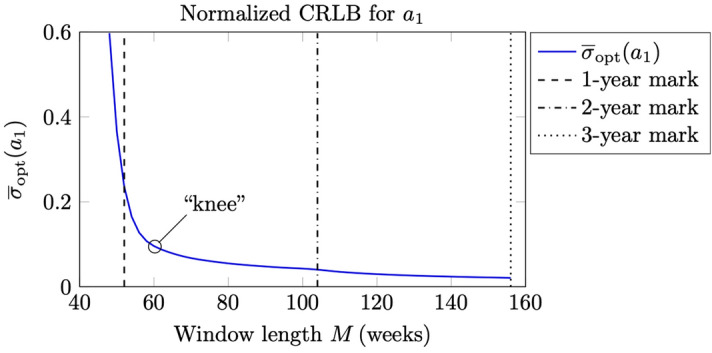
The relative error in the estimation of the rate of change in the trend of the Keeling curve (parameter $a_1$), plotted here as an L-curve with respect to the window length *M*.

To obtain a unitless measure of the estimation errors we consider the following normalized Cramér–Rao lower bounds (relative errors)

19 $$\begin{aligned} {\overline{\sigma }}_{\text {opt}}(a_0)&= \frac{\sqrt{{\text {CRB}}\{a_0\}}}{{\text {mean}}\{{\widehat{a}}_0\}}, \end{aligned}$$

20 $$\begin{aligned} {\overline{\sigma }}_{\text {opt}}(a_1)&= \frac{\sqrt{{\text {CRB}}\{a_1\}}}{{\text {mean}}\{{\widehat{a}}_1\}}, \end{aligned}$$

where ${\text {CRB}}\{a_0\}$ and ${\text {CRB}}\{a_1\}$ are given by (12) and (13), and where ${\text {mean}}\{{\widehat{a}}_0\}$ and ${\text {mean}}\{{\widehat{a}}_1\}$ are the respective mean values of the estimated parameters for the whole time interval of interest. In Fig. 6 is shown the relative error ${\overline{\sigma }}_{\text {opt}}(a_1)$ plotted as an L-curve with respect to the window length *M*. All the weekly data between 1974 and 2020 have been used to obtain the mean value, and as the variance of the noise we have employed the estimate $\widehat{\sigma ^2}=0.145$ ppm^2^.

**Figure 7:**
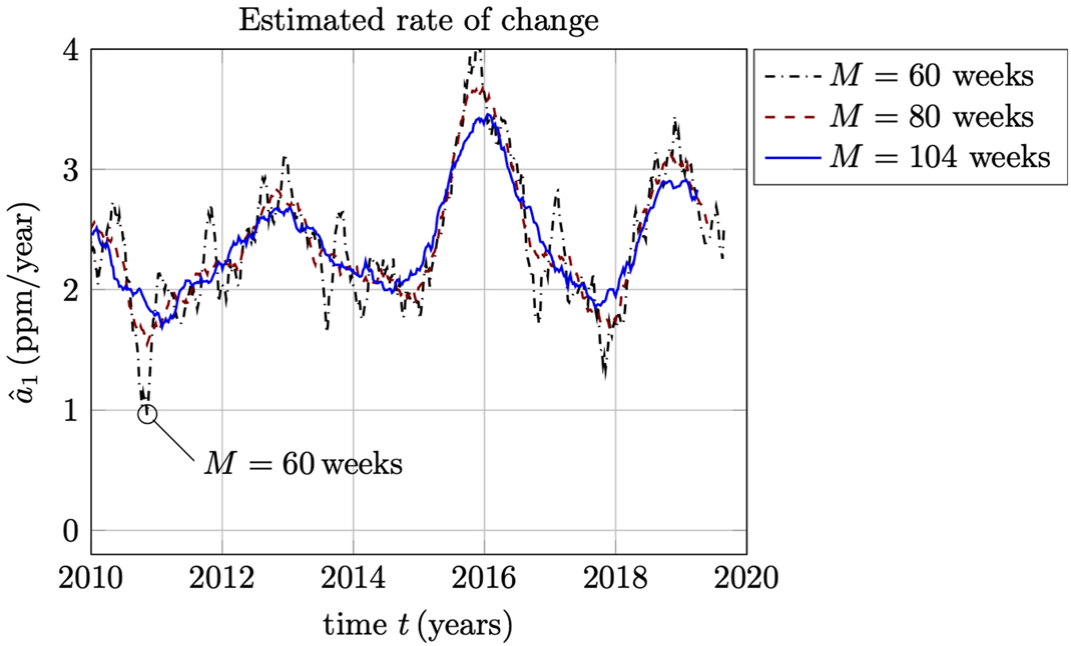
Estimated rate of change ${\widehat{a}}_1$ for data windows with length $M=60$, $M=80$ and $M=104$ weeks. Notice the unstable behavior when the window length approaches the “knee” of the L-curve around $M=60$, as seen in Fig. 6.

The relative error in the estimate of the rate of change (the derivative) $a_1$ is more than 2 orders of magnitude ( $100\times$) larger than the relative error in the estimate of the trend itself, $a_0$, which is not shown here, cf. also^9^. Nevertheless, provided that the window length *M* (the regularizing parameter) is large enough, we can indeed have a stable estimate of the derivative $a_1$. The relative errors shown in Fig. 6 agree rather well with the qualitative behavior of the estimate ${\widehat{a}}_1$, as is illustrated in Fig. 7. At $M=60$ weeks or less, the “knee” of the L-curve is approached and the estimation becomes unstable. With a short-time estimate of the rate of change based on a 2-year window ( $M=104$ weeks) we have a stable estimate with a relative error less than 5%. It is noted that the average slope is about 1.8 ppm/year, so that 5% corresponds to about 0.09 ppm/year. For even larger window lengths, the estimates get more and more smoothed out, as would be expected.

## The short-time rate of change in context

It may be of interest to compare or to correlate the estimated rate of change in the trend of the Keeling curve with natural phenomena as well as the major anthropogenic activities. Of course, any conclusions based on such a comparison must be treated with great care considering the vast complexity of the Earth system. Nevertheless, to demonstrate the application of the estimation of trends in $\text {CO}_2$ data, it is worthwhile to put some of these global events together and to discuss their possible interpretations.

**Figure 8:**
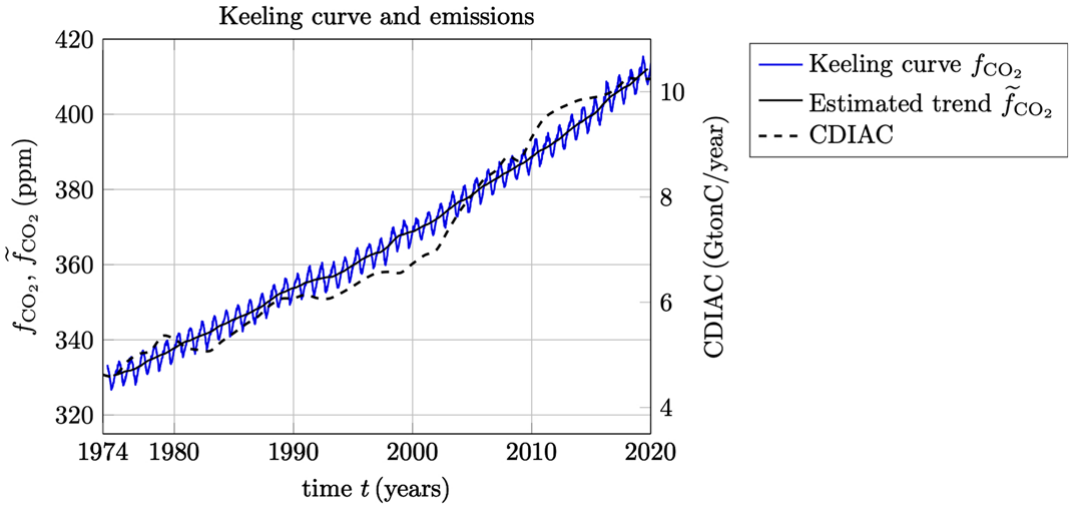
The Keeling curve $f_{{\text {CO}}_2}$ and the estimated trend ${\widetilde{f}}_{{\text {CO}}_2}$ (in ${\text {ppm}}$ on the left *y*-axis), in a comparison with global ${\text {CO}}_2$ emissions from fossil-fuel burning according to CDIAC (in ${\text {GtonC/year}}$ on the right *y*-axis).

**Figure 9:**
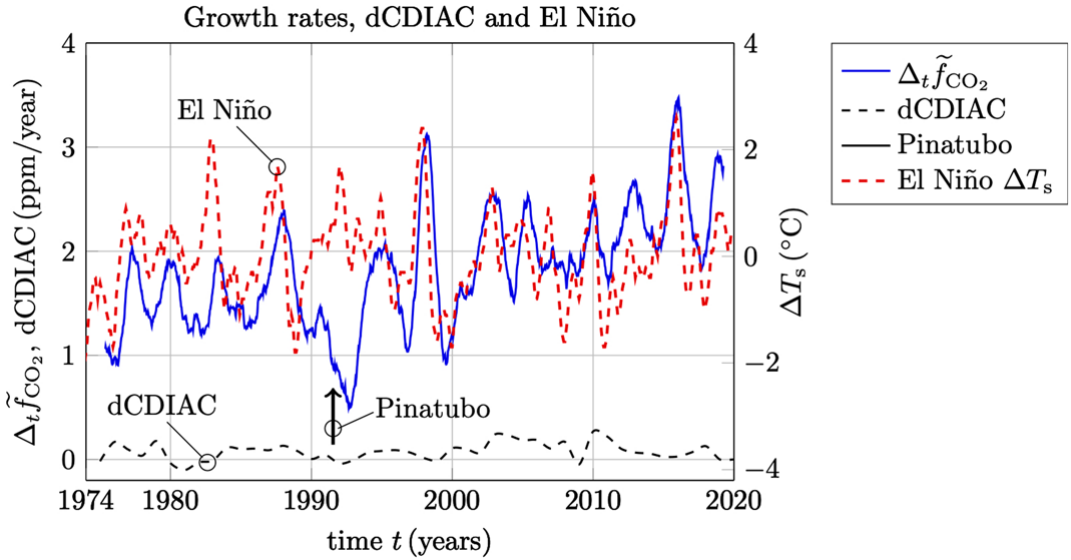
The estimate $\Delta _t {\widetilde{f}}_{{\text {CO}}_2}$ of the rate of change in the trend of the Keeling curve in a comparison with year-to-year differences dCDIAC of the CDIAC data rescaled to the units of ppm/year shown on the left *y*-axis. The El Niño related SSTs are plotted as $\Delta T_{\text {s}}$ in °C shown on the right *y*-axis. The time of the Pinatubo eruption is indicated with an arrow.

In Fig. 8 is shown the Keeling curve and its estimated trend over the period 1974–2020 plotted in ppm ${\text {CO}}_2$ on the left *y*-axis, together with estimated values of global ${\text {CO}}_2$ emissions from fossil-fuel burning according to CDIAC^15^ which is presented here in units of ${\text {GtonC/year}}$ (Gigaton carbon per year) on the right *y*-axis. It is noted that the last update of CDIAC is from 2014^15^. The values for 2015–2018 have been extrapolated from BP review by multiplying the increase factors from BP review to the carbon numbers in CDIAC. The values for 2019–2020 are assumed to be constant. The plot in Fig. 8 can serve to illustrate the present day non-sustainability of fossil-fuel burning, which by an overwhelming consensus among climate experts in the world is the main cause of global warming and climate change^5^. Many people are confused about emissions and ${\text {CO}}_2$. When emissions become smaller they expect ${\text {CO}}_2$ to go down. Chemical destruction in the atmosphere occurs for many chemicals on different time scales but for ${\text {CO}}_2$ it is very close to zero. Therefore, the ${\text {CO}}_2$ level in the atmosphere is the integral of all past emissions and removals^16^. That is why the trend of ${\text {CO}}_2$ is so smooth compared to the emissions history, why the current emissions slowdown is so hard to see, and why the diagnosis of changes of sources/sinks depends on the reliable detection of very small spatial/temporal differences of atmospheric ${\text {CO}}_2$.

In Fig. 9 is shown the estimated rate of change in the trend of the Keeling curve $\Delta _t {\widetilde{f}}_{{\text {CO}}_2}$ defined in (2) in a comparison with some major global events. The growth rates are plotted in ${\text {ppm/year}}$ shown on the left *y*-axis. The NOAA growth rates of Fig. 3 are not shown here as they are almost indistinguishable from the estimates $\Delta _t {\widetilde{f}}_{{\text {CO}}_2}$ in this plot. The historical El Niño/La Niña episodes are shown as Sea Surface Temperature (SST) anomalies in the Niño 3.4 region (5° N–5° S, 120°–170° W), see^17^. These SSTs are plotted here as $\Delta T_{\text {s}}$ in °C shown on the right *y*-axis of the figure. In the same figure, we have also plotted year-to-year differences (dCDIAC) of the CDIAC data that was shown in Fig. 8, and which has been rescaled here to units of ${\text {ppm/year}}$. It takes 2.124 ${\text {GtonC}}$ to change the entire atmosphere by 1 ${\text {ppm}}$^18,19^. If the emission change is for 1 year, then there has not been enough time for the anomaly to spread uniformly through the atmosphere. The Southern Hemisphere (SH) has only partially caught up with the NH where the emissions change originated, nor has the stratosphere. So, in 1 year the emissions are effectively diluted into 70–80 $\%$ of the atmosphere, which means that 1.5–1.7 ${\text {GtonC}}$ will cause a 1 ${\text {ppm}}$ change in the NH troposphere. Hence, we have normalized the year-to-year differences of CDIAC data with a factor of 1.6 to yield the differences in units of ${\text {ppm/year}}$. Finally, we have indicated the time of the Pinatubo eruption in june 1991, which is believed to have caused a climate anomaly lasting for about 2 years. It should be emphasized that the El Niño and the Pinatubo climate anomalies have been studied extensively by many researchers, and that they are considered here merely as a demonstration and a confirmation that the estimated growth rates are reliable.

The changes seen in dCDIAC seem to correlate somewhat with the major economic recessions in 1980–1983, 1990–1993, 2001–2002 and 2008–2009. However, the corresponding changes in dCDIAC are too small to be reliably connected to the rather strong fluctuations that are seen in the estimated growth rates $\Delta _t {\widetilde{f}}_{{\text {CO}}_2}$ over this period. On the other hand, the timings of the El Niño SST anomalies are clearly correlated with the observed changes in the estimated growth rates, except for the times around the climate anomaly followed by the Pinatubo eruption. The years 1992 and 1993 following Pinatubo were relatively cool, which is likely to have reduced respiration from plants and soils, thus lowering atmospheric ${\text {CO}}_2$. Furthermore, the increased portion of diffuse (scattered) light enhanced photosynthesis by reducing self-shading in plant canopies, enhancing ${\text {CO}}_2$ removal from the atmosphere^20^. It may also be noted that the eruption of Pinatubo was estimated to produce some extra 50 ${\text {Mtons}}$ of ${\text {CO}}_2$ into the atmosphere over a very short time. However, this corresponds to a change of only $0.05\times 12/44/1.6=0.0085$ ${\text {ppm}}$ in the atmosphere, which is too small to be observable (the ratio 12/44 corresponds to the molecular weights of carbon to carbon dioxide).

**Figure 10:**
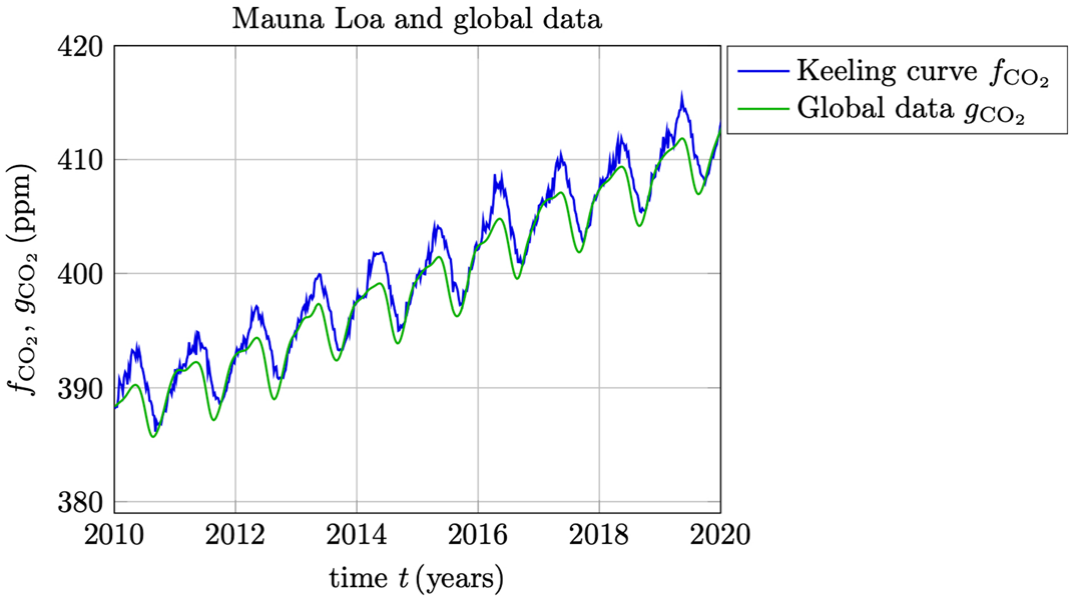
Weekly averaged data ${f}_{{\text {CO}}_2}$ from the Mauna Loa station (the Keeling curve) in a comparison with globally (and weekly) averaged data ${g}_{{\text {CO}}_2}$ from the four NOAA/GML baseline observatories.

**Figure 11:**
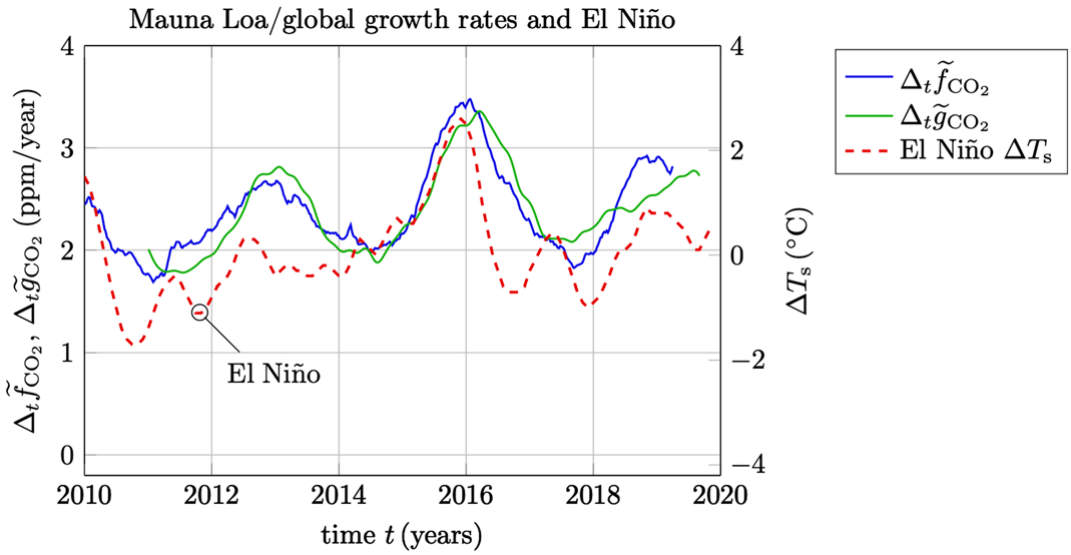
A comparison between the estimated growth rates $\Delta _t {\widetilde{f}}_{{\text {CO}}_2}$ and $\Delta _t {\widetilde{g}}_{{\text {CO}}_2}$ based on the Mauna Loa site and the global data, respectively. The El Niño related SSTs are plotted as $\Delta T_{\text {s}}$ shown on the right *y*-axis. Notice the slight delay of the global response $\Delta _t {\widetilde{g}}_{{\text {CO}}_2}$ in comparison to the local response $\Delta _t {\widetilde{f}}_{{\text {CO}}_2}$.

It is observed that the statistical analysis presented in this paper is restricted to one station only, the MLO in Hawaii. The main reason for choosing this particular station is the uniqueness of the extensive record of weekly data that is available since 1974, and which have made it possible to assess the Gaussian statistics with very high precision. Nevertheless, the simple estimation strategy expressed in (1) and (2) is readily applicable to globally averaged multistation data such as the daily data that is available from the NOAA/GML baseline observatories in Barrow (Alaska), Mauna Loa (Hawaii), American Samoa and the South Pole (Antarctica) with data from 2010 to 2020, cf.^21^. In Fig. 10 is shown a comparison between the weekly averages of MLO data ${f}_{{\text {CO}}_2}$ and the corresponding globally (and weekly) averaged data from the four NOAA/GML baseline observatories which is denoted here by ${g}_{{\text {CO}}_2}$. In Fig. 11 is shown the corresponding growth rate estimates (2) denoted $\Delta _t {\widetilde{f}}_{{\text {CO}}_2}$ and $\Delta _t {\widetilde{g}}_{{\text {CO}}_2}$, respectively, and where the El Niño related SSTs are also shown in the same figure. As can be seen in Fig. 11, the local (MLO) and the global growth rates behave very similar and deviate maximally with about 0.5 ${\text {ppm/year}}$ over this period. In Fig. 10 it can furthermore be seen from the timing of the annual variability (seasonal curves) that the global cycle is somewhat ahead of the local cycle at MLO. More interestingly, however, is that in Fig. 11 we can see that the growth rate of the global trend seems to be slightly delayed with respect to the growth rate of the local trend at MLO. This makes sense considering that the time-constants associated with the response to the El Niño stimulated phenomena is expected to be larger for global growth rates in comparison to the local responses.

It may finally be of interest to consider the potential impact that the COVID-19 crisis may have on the present and future ${\text {CO}}_2$ readings. According to the International Energy Agency IEA, the global ${\text {CO}}_2$ emissions are expected to decline to reach 30.6 ${\text {Gton}}$ ${\text {CO}}_2$ for 2020, almost 8 $\%$ lower than in 2019. This means an abrupt change of 2.66 ${\text {Gton}}$ less ${\text {CO}}_2$ in comparison to the previous year, or 0.45 ${\text {ppm}}$ using the conversion factors 3.667 for ${\text {C}}\rightarrow {\text {CO}}_2$ and 1.6 for ${\text {ppm}}\rightarrow {\text {GtonC}}$. In principle, a 0.45 ${\text {ppm/year}}$ drop in the growth rate should be clearly visible in the Keeling curve from the point of view of the 5 $\%$ estimation accuracy that is presented here. However, as illustrated in Figs. 9 and 11 one must also consider the variability in the El Niño or other climate related phenomena that may cause much stronger fluctuations in the estimated growth rate. In any case, due to the centered 2-year data window which is required in order to obtain a reliable estimate of the rate of change, we can conclude that we will have to wait until 2021 to see any conclusive effects of the COVID-19 outbreak in the atmospheric ${\text {CO}}_2$ data.

## Summary and conclusion

We have proposed a simple strategy for estimating the short-time rate of change in the trend of the Keeling curve. This estimate is based on a centered 1-year sliding average to estimate the trend, and a corresponding centered 2-year sliding data window with differencing to determine its rate of change. To validate this estimator, we have compared it to a more sophisticated regression analysis based on a combined Taylor and Fourier series expansion and found a very good agreement based on 3 Taylor coefficients, 8 Fourier series coefficients (4 yearly harmonics) and a 2-year data window to determine the parameters. A statistical analysis based on weekly data for the years 1974–2020 has shown that the model errors can be considered to be uncorrelated and identically Gaussian distributed. The Gaussian assumption justifies the use of standard formulas for Maximum Likelihood (ML) estimation, Fisher information and the related Cramér-Rao Lower Bounds (CRLB). An L-curve technique based on the CRLB has been used to study the accuracy and stability of the regression analysis. With the regression model that has been studied here, it is found that the limit of stable inversion for the rate of change in the trend of the Keeling curve is about 50–60 weeks, and that the 2-year window of 104 weeks yields a relative error less than 5 $\%$. Hence, to obtain a stable, reliable and readily accessible estimate, we propose to use the simple strategy based on a 2-year data window with differencing, as described above. As a confirmation of the method we have found a very good agreement with the trend analyses performed by NOAA. As an illustration of the method, we have demonstrated how the growth rates are visibly correlated with the El Niño climate phenomenon. And finally, as an interesting topic for future research, we would propose an in-depth study of multistation data in order to determine the statistical accuracy for the joint estimation of global growth rates.

## References

[1] Thoning KW, Tans PP, Komhyr WD (1989). Atmospheric carbon dioxide at Manua Loa observatory 2. Analysis of the NOAA GMCC data, 1974–1985. J. Geophys. Res..

[2] Lüthi D (2008). High-resolution carbon dioxide concentration record 650,000–800,000 years before present. Nature.

[3] Etheridge DM (1996). Natural and anthropogenic changes in atmospheric $$\text{CO}_2$$ over the last 1000 years from air in Antarctic ice and firn. J. Geophys. Res. Atmos..

[4] Rubino M (2013). A revised 1000 year atmospheric $$\delta ^{13}\text{ C }$$- $$\text{ CO}_2$$ record from Law Dome and South Pole, Antarctica. J. Geophys. Res. Atmos..

[5] Stocker T (2013). IPCC, 2013: Climate Change 2013: The Physical Science. Basis Contribution of Working Group I to the Fifth Assessment Report of the Intergovernmental Panel on Climate Change.

[6] Harris DC (2010). Charles David Keeling and the story of atmospheric $$\text{ CO}_2$$ measurements. Anal. Chem..

[7] Curve Fitting Methods Applied to Time Series in NOAA/ESRL/GMD. (Accessed 10 May 2020); https://www.esrl.noaa.gov/gmd/ccgg/mbl/crvfit/.

[8] Dr. Pieter Tans, NOAA/ESRL (www.esrl.noaa.gov/gmd/ccgg/trends/) and Dr. Ralph Keeling, Scripps Institution of Oceanography (www.scrippsco2.ucsd.edu/). Accessed 10 May 2020.

[9] Naeem, M. F. *Analysis of an Ill-posed Problem of Estimating the Trend Derivative Using Maximum Likelihood Estimation and the Cramér-Rao Lower Bound*. Master’s thesis, Linnæus University, 351 95 Växjö, Sweden. (2020). URN: urn:nbn:se:lnu:diva-95163.

[10] Kirsch A (1996). An Introduction to the Mathematical Theory of Inverse Problems.

[11] Hansen PC (1992). Analysis of discrete ill-posed problems by means of the L-curve. SIAM Rev..

[12] Hansen PC (2010). Discrete Inverse Problems: Insight and Algorithms.

[13] Kay SM (1993). Fundamentals of Statistical Signal Processing, Estimation Theory.

[14] Proakis JG, Manolakis DG (2007). Digital Signal Processing.

[15] Boden T, Marland G, Andres R (2017). Global, Regional, and National Fossil-Fuel CO2 Emissions.

[16] Tans P (2009). An accounting of the observed increase in oceanic and atmospheric $$\text{ CO}_2$$ and an outlook for the future. Oceanography.

[17] Historical El Niño/La Niña episodes (1950–present). United States Climate Prediction Center (accessed 13 May, 2020). https://origin.cpc.ncep.noaa.gov/products/analysis_monitoring/ensostuff/ONI_v5.php.

[18] Carbon Dioxide Information Analysis Center—Conversion Tables. (Accessed 10 May 2020); https://cdiac.ess-dive.lbl.gov/pns/convert.html.

[19] Glossary: Carbon Dioxide and Climate. Environmental Sciences Division Publication No. 3532 (Carbon Dioxide Information Analysis Center, Oak Ridge National Laboratory, U.S. Department of Energy, Oak Ridge, 1990).

[20] Gu L (2003). Response of a deciduous forest to the Mount Pinatubo eruption: enhanced photosynthesis. Science.

[21] Ed Dlugokencky and Pieter Tans, NOAA/GML. (Accessed 10 May 2020); www.esrl.noaa.gov/gmd/ccgg/trends/.

